# Alpha-Tocopherol Counteracts the Cytotoxicity Induced by Ochratoxin A in Primary Porcine Fibroblasts

**DOI:** 10.3390/toxins2061265

**Published:** 2010-06-01

**Authors:** Eleonora Fusi, Raffaella Rebucci, Chiara Pecorini, Anna Campagnoli, Luciano Pinotti, Francesca Saccone, Federica Cheli, Stig Purup, Kristen Sejrsen, Antonella Baldi

**Affiliations:** 1 Università degli Studi di Milano, Department of Veterinary Science and Technology for Food Safety, Via Trentacoste 2, I-20134 Milan, Italy; Email: raffaella.rebucci@unimi.it (R.R.); chiara.pecorini@unimi.it (C.P.); luciano.pinotti@unimi.it (L.P.); francesca.saccone@unimi.it (F.S.); federica.cheli@unimi.it (F.C.); antonella.baldi@unimi.it (A.B.); 2 Università Telematica San Raffaele, Roma, Via di Val Cannuta, 247, 00166 Roma, Italy; Email: anna.campagnoli@docenti.uni-tel.it (A.C.); 3 Department of Animal Health and Bioscience University of Aarhus, Research Centre Foulum, P.O. Box 50, DK-8830 Tjele, Denmark; Email: stig.purup@agrsci.dk (S.P.); kristen.sejrsen@agrsci.dk (K.S.)

**Keywords:** ochratoxin A, α-tocopherol, DNA damage, fibroblasts, swine

## Abstract

The aims of the current study were to determine the half-lethal concentration of ochratoxin A (OTA) as well as the levels of lactate dehydrogenase release and DNA fragmentation induced by OTA in primary porcine fibroblasts, and to examine the role of α-tocopherol in counteracting its toxicity. Cells showed a dose-, time- and origin-dependent (ear *vs.* embryo) sensitivity to ochratoxin A. Pre-incubation for 3 h with 1 nM α-tocopherol significantly (P < 0.01) reduced OTA cytotoxicity, lactate dehydrogenase release and DNA damage in both fibroblast cultures. These findings indicate that α-tocopherol supplementation may counteract short-term OTA toxicity, supporting its defensive role in the cell membrane.

## Abbreviations

LC_50_: half-lethal concentration
LDH: lactate dehydrogenase
MTT: 3-(4,5-dimethylthiazol-2-yl)-2,5-diphenyltetrazoliumbromide
OTA: ochratoxin A
TUNEL: Terminal deoxynucleotidyl transferase dUTP nick end labeling

## 1. Introduction

Ochratoxin A (OTA) is a mycotoxin produced by *Aspergillus* and *Penicillum* species. It occurs in several agricultural products and causes diseases both in humans and animals [1]. OTA has been implicated in renal and hepatic toxicity, neurotoxicity, teratogenicity, and immunotoxicity [2], and it is considered by the International Agency for Research on Cancer as possibly carcinogenic (group 2B) to humans [3], making consistent exposure to OTA a cause of serious concern.

The scientific panel on contaminants in the food chain of the European Food Safety Authority released an opinion related to OTA in food [4], in which it summarized the major information on OTA related to human health but also indicated the susceptibility of pigs and other animals to OTA. In porcine species, OTA is responsible for acute, subchronic and chronic intoxications, and the effects, correlated with the latter two, are of major concern for financial losses in the agriculture and food industry [5]. The main clinical patterns associated with OTA intoxication in swine are impaired renal function, depression, anorexia, decreased weight gain and productivity [6].

At the cellular level, OTA toxicity involves various mechanisms of action: lipid peroxidation, disruption of calcium homeostasis, inhibition of protein synthesis, mitochondrial respiration, and DNA damage [7]. The toxicological effects of OTA depend on the duration and concentration of exposure [5]. OTA can act in different ways: it can express its toxicity directly or by indirect mechanisms, such as by inducing cytotoxicity, oxidative cell damage and increased cell injury [8]. Previous reports [2,5,6,7,8,9,10,11] indicate that the OTA toxicity and DNA damage, measured *in vivo* and *in vitro*, are most likely attributable not only to cellular oxidative damage mediated by lipid peroxidation, but also to direct genotoxic effect. However, the molecular mechanism involved in the apoptotic or antiproliferative effects of OTA is still unclear. In particular, O’Brien and Dietrich [5], in their review on OTA, highlighted its questionable role as a pro-apoptotic or pro-cytotoxic agent. Considering that induction of cell death is a process involving many factors, such as substance, dose/time exposure and the cellular *in vitro* models investigated, OTA could be responsible for an apoptotic or a necrotic process. Understanding the molecular mechanism of action of OTA is essential for improving the toxicity-reducing countermeasures applied.

α-Tocopherol is a member of the vitamin E compound group that has several biological roles [12,13]. Vitamin E is a potent antioxidant; its function as a peroxyl radical scavenger that terminates chain reactions is well documented [14,15]. The beneficial effects of vitamin E, particularly of α-tocopherol, include its position and movement within the cellular membrane, its ability to donate H atoms, as well as the efficiency of tocopheroxyl radical recycling by cytosolic reductants (*i.e.*, Antioxidant network vitamin E–vitamin C–Glutathione (GSH)–thiol redox cycle) [16,17]. In light of these properties, the use of antioxidant compounds as counteracting agents against both the oxidative damage induced by OTA through membrane lipid peroxidation and OTA genotoxicity have been reported by several authors [18,19]. *In vivo*, it has been demonstrated that vitamin E decreases OTA genotoxicity [20], while our previous *in vitro* studies indicated that α-tocopherol has protective activities against OTA, reducing ROS production in established cell lines [18,21].

The primary aim of this study was to determine the toxic effects of OTA in primary porcine fibroblast cell cultures by the MTT assay, LDH release, DNA fragmentation and TUNEL stain. Further, we aimed to determine the contribution of α-tocopherol in counteracting the cytotoxicity and DNA damage induced by OTA in the same *in vitro* model.

## 2. Results and Discussion

### 2.1. Cytotoxic effect and LDH release induced by ochratoxin A

We first investigated the LC_50_ of OTA after 24 h and 48 h of treatment in ear and embryo porcine fibroblasts and found that the LC_50_ differed between the two cell types. At all incubation times, the fibroblasts derived from ear were the most sensitive to OTA cytotoxicity (LC_50_ = 0.93 μg/mL after 24 h; LC_50_ = 0.92 μg/mL after 48 h), while fibroblasts isolated from the embryo showed a time-dependent sensitivity (LC_50_ = 4.24 μg/mL after 24 h, 2.34 μg/mL after 48 h). Previous studies have shown different cytotoxic responses to *in vitro* OTA challenges in different cell lines [18]. Our results confirm that the origin of the cells could explain the response to OTA stimuli, as reported by several groups [22,23]. To date, most of the studies have been conducted using epithelial cells originated from several mammalian species, but only human fibroblasts were used in some studies [19,22].

**Figure 1:**
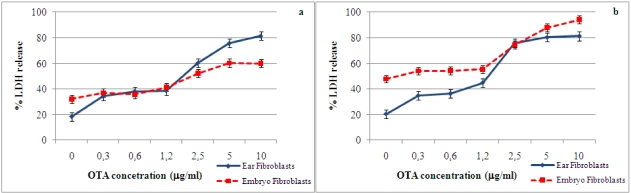
Concentration-dependent release of LDH into culture media by primary porcine ear and embryo fibroblasts after 24 h (**a**) and 48 h (**b**) exposure to OTA concentrations. Values are means, with standard errors of the mean represented by vertical bars.

Figure 1 shows the data on LDH release by primary porcine ear and embryo fibroblasts in the presence of several concentrations of OTA at 24 and 48 h of incubation. In both cell types, LDH release increased significantly (P < 0.01) at OTA concentrations above 2.5 μg/mL after 24 and 48 h of incubation. Schwerdt *et al.* [22], in their studies on the long-term effects of OTA on primary fibroblasts, indicated that LDH release in the media increased only after five days of exposure to OTA, while Russo *et al.* [19] reported a significant LDH release after 72 h of OTA treatment. However, in our study, after only 24 h of incubation, the fibroblast cultures showed considerable LDH release when OTA was present at concentrations similar to the doses used by Russo *et al.* [19], indicating early cellular membrane damage.

### 2.2. Detection and quantification of DNA damage induced by ochratoxin A

**Figure 2:**
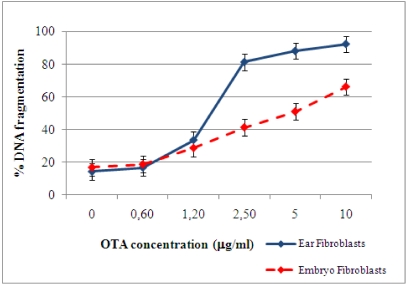
DNA fragmentation in primary fibroblasts 24 h post–OTA stimulation.

OTA was able to induce DNA fragmentation in both primary porcine fibroblast cultures, as measured by a diphenylamine assay. After 24 h of incubation, in the absence of OTA (control), 14% DNA fragmentation in both primary cultures occurred. A dose-dependent fragmentation in fibroblast cultures, due to the exposure to increasing concentrations of OTA, was found (Figure 2). At 0.6 μg/mL of OTA, the DNA fragmentation percentage of both types of fibroblasts were similar to control levels. Exposure to high OTA concentrations (2.5, 5, or 10 μg/mL) led to increasing percentages of DNA fragmentation in the two types of cells. At 10 μg/mL of OTA, DNA fragmentation was 92% in fibroblasts isolated from ear and 66% in embryonic fibroblasts. These results confirm the different sensitivities of fibroblasts to OTA-induced cell damage. Russo *et al.* [19] report that human fibroblast cultures show DNA damage after 72 h of OTA exposure at high concentration. However, OTA concentrations lower than the dose used by Russo *et al.* [19] revealed that these damages occurred earlier and were present after 24 h of OTA exposure. The different origins of the cells (human *vs.* porcine) could explain the difference in sensitivity. Moreover, as indicated by O’Brien *et al.* [24], the cellular response to OTA toxicity related not only to the amount of the mycotoxin that gained access to the cells but also to the individual cell tolerance to OTA load. 

**Figure 3:**
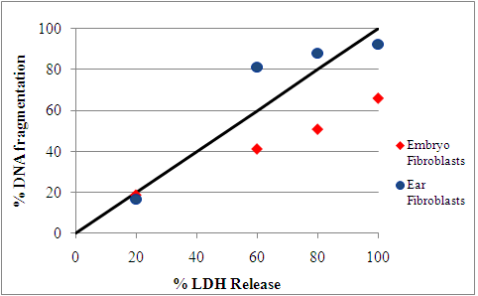
Comparison of DNA fragmentation and LDH release in fibroblasts cultures after 24 h of exposure to 0.6, 2.5, 5, or 10 μg/mL OTA. The solid line indicates an equal percentage of DNA fragmentation and LDH release. During necrosis, the experimental values should be close to this line [25]. In the presence of the selected OTA concentrations, in both cell cultures the experimental values indicated proportional changes in DNA fragmentation and LDH release.

Cell death may occur by several mechanisms. It is well known that LDH release is a marker of cellular membrane damage, while DNA fragmentation measurements give an indication of the percentage of fragmented DNA out of the total nuclear DNA of cultured cells. A comparison of DNA fragmentation with LDH release after 24 h of OTA exposure is shown in Figure 3. After 24 h exposure to 0.6, 5, or 10 μg/mL OTA in both fibroblast cultures, we observed that the changes in DNA fragmentation and LDH release were almost proportional. Based on these data, we suggest that the cell death process induced by OTA involved both cellular and DNA damage. Gekle *et al.* [25], showed that OTA exposure induces apoptosis and necrosis in the MDCK-C7 and MDCK-C11 cells, respectively, as indicated by comparing DNA fragmentation with LDH release. 

### 2.3. Detection of DNA damage by TUNEL assay

**Figure 4:**
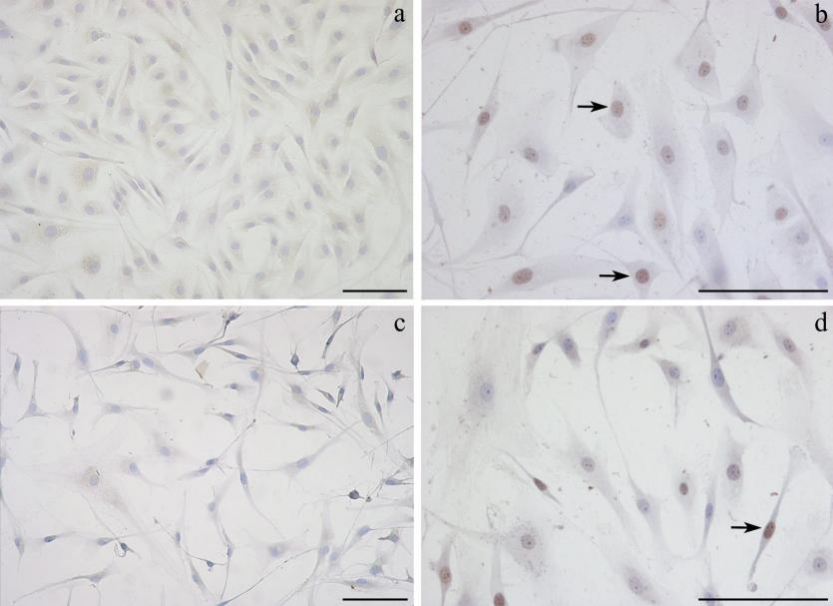
Representative photographs of selected fields of TUNEL-stained ear fibroblasts are shown. The TUNEL-positive nuclei, indicating apoptotic cells, are stained brown (black arrows), while the vital nuclei are stained violet (haematoxylin). (**a**) Ear fibroblasts in culture for 24 h (no OTA). (**b**) Ear fibroblasts treated with 0.6 μg/mL OTA for 24 h. (**c**) Ear fibroblasts treated with 1.25 μg/mL OTA for 24 h. (**d**) Ear fibroblasts treated with 1 nM α-tocopherol and 0.6 μg/mL OTA for 24 h. Bars = 200 μm.

In a series of experiments, we examined the effect of OTA exposure on fibroblast cultures using the TUNEL assay. Figure 4 shows representative photos of ear fibroblast morphology and nuclear stains in cells maintained in culture and exposed to OTA for 24 h. In the absence of OTA, the uniformity of monolayers, the typical fibroblast shape and cell-cell interactions in cultures were evident in fibroblasts from both sources tested. Only 4% and 6% of nuclei were apoptotic in cultures of primary ear and embryonic fibroblasts, respectively. The percentage of dark-brown apoptotic nuclei in primary fibroblasts, isolated from ear and embryo, co-incubated for 24 h with 0.6 μg/mL of OTA, were 32% and 16%, respectively. In both ear and embryo fibroblast cultures after 24 of OTA incubation above LC_50_, monolayers were completely destroyed and cell debris were evident in all the microscopic fields.

Although studies conducted by several groups [10,22,25] indicate the apoptotic pathway as the means by which OTA induces toxicity *in vivo* and *in vitro*, the determination of predominant cell death pathways depends on several conditions, such as the experimental model, the dose of toxin and the duration of the exposure. The mechanisms and morphologies of apoptosis and necrosis are different, but there is an overlap between these two processes, in what has been called “necrosis–apoptosis continuum” [26]. In fact, it is not simple to distinguish the predominant process, as apoptosis and necrosis can occur simultaneously [27]. Understanding the evolution of cell death in cellular models is critical. Therefore, once again, it is important not only to consider the concentration and duration of toxin exposure and the cell type, but also to employ multiple endpoint assays [24].

### 2.4. Effect of α-tocopherol on ochratoxin A–induced toxicity

The inhibitory effects of α-tocopherol on OTA cytotoxicity were evaluated using several toxicity assays: MTT test, LDH release, DNA fragmentation and TUNEL assays.

Pre-treatment with α-tocopherol (1 nM) for 3 h, followed by OTA exposure for 24 h, significantly (P < 0.01) reduced the loss of cell viability induced by OTA in both *in vitro* models, as determined by the MTT test: by 17.5% in ear fibroblasts exposed to 0.3 μg/mL OTA and by 14.7% in embryonic fibroblasts exposed to 0.6 μg/mL OTA. Treatment with 1 μM of antioxidant solution did not affect OTA toxicity. After 48 h, α-tocopherol at both concentrations was unable to counteract the OTA-induced cytotoxicity in either cell type.

**Figure 5:**
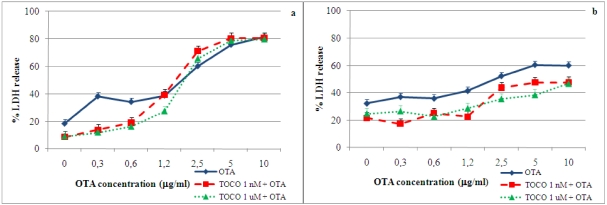
Effect of α-tocopherol on OTA-induced LDH release in primary porcine fibroblasts at 24 h. Values are means, with standard errors of the mean represented by vertical bars. (**a**) Ear fibroblasts. (**b**) Embryonic fibroblasts.

The data on LDH release induced by OTA after 3 h pre-incubation with 1 nM or 1 μM α-tocopherol in fibroblast cultures are shown in Figure 5. At 24 h of incubation, in ear fibroblasts, α-tocopherol at both concentrations significantly (P < 0.01) decreased LDH release when OTA was present at 0.3–0.6 μg/mL. In embryonic fibroblasts at both concentrations of antioxidant, LDH release in the presence of all OTA concentrations used was significantly reduced (P < 0.05) compared to control cultures (OTA only). After 48 h of co-incubation with tocopherol and OTA, no difference in LDH release in either cell type was detected. These data confirm that the origin of the cells can not only explain the response to OTA stimuli, but also the sensitivity to antioxidant response.

**Figure 6:**
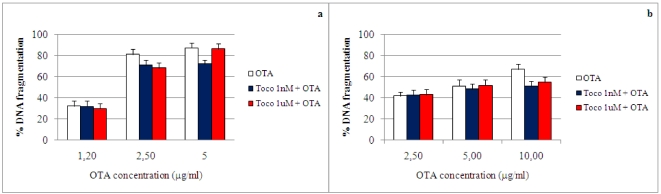
Effect of α-tocopherol on OTA-induced DNA fragmentation in primary porcine fibroblasts at 24 h. Values are means, with standard errors of the means represented by vertical bars. (**a**) Ear fibroblasts. (**b**) Embryo fibroblasts.

The effects of 3 h pre-incubation with 1 nM or 1 μM α-tocopherol on OTA-induced DNA fragmentation in primary fibroblasts is shown in Figure 6. Treatment with 1 nM α-tocopherol resulted in a significant (P < 0.05) decrease in DNA fragmentation compared with control incubation (OTA only). This decrease was 15% in the presence of 5 μg/mL of OTA in ear fibroblasts and 16% in the presence of 10 μg/mL of OTA in embryonic fibroblasts.

The effects of α-tocopherol pre-treatment on DNA fragmentation induced by OTA were evaluated by TUNEL assay. In ear fibroblasts α-tocopherol pre-treatment (1 nM or 1 μM) did not significantly reduce the percentage of apoptotic nuclei by 12% and 9%, respectively. A representative photograph of ear fibroblasts pre-incubated with α-tocopherol and co-incubated with 0.6 μg/mL OTA for 24 h is shown in Figure 4(d). In embryonic fibroblasts, α-tocopherol pre-treatment (1 nM or 1 μM) insignificantly decreased the percentage of apoptotic nuclei in the presence of 0.6 μg/mL OTA by 11% and 8%, respectively. α-tocopherol was not able to reduce the damage to nuclei when OTA concentrations were above LC_50_ (embryonic: 5 μg/mL OTA; ear: 1.2 μg/mL OTA), since the cells of treated monolayers detached, and cellular debris floated in the observed fields (data not shown).

Taken together, our results indicate that α-tocopherol counteracts the toxicity of the mycotoxin OTA. In particular, the pre-incubation with α-tocopherol reduced LDH release and DNA damage in both fibroblast cultures, thereby preserving the integrity of the cells. It is well known that α-tocopherol is fundamental to cellular defence mechanisms against endogenous and exogenous oxidant agents [15]. This compound is a peroxyl radical scavenger that terminates the free radical chain reaction [16], and its hydrophobic nature stabilises its position in the phospholipid bilayer. In fact, in the core of the cellular membrane, as described by Traber and Atkinson [16], the antioxidant characteristics of α-tocopherol are due to its H atom donating ability, its position and movements in the cellular membrane, and its activity in cytosolic reduction reactions that recycle tocopheroxyl radicals, thus preventing lipid peroxidation. 

As demonstrated by Baldi *et al.* [18], in MDCK and BME-UV1 cell lines, the cytotoxicity and ROS production induced by OTA are reduced by antioxidant pre-treatments. In particular, α-tocopherol significantly reduces OTA-induced ROS production. This inhibition is concentration-dependent: treatment with a higher concentration (10 mM) results in a significantly greater degree of protection against ROS production. OTA causes lipid peroxidation and free radical formation in mammalian cells. The oxidative metabolism sustained by enzymes such as cytochrome P450 enzymes and enzymes with peroxidase activities, is responsible for OTA biotransformation and subsequent ROS production [28]. The OTA toxicity reduction is due to the scavenging of lipid hydroperoxil radicals by vitamin E. This is due to the increase in the activity of the glutathione peroxidase, which utilizes GSH for catalyzing the reduction of hydroperoxides [20].

Moreover, in BME-UV1 cells, α-tocopherol at two different concentrations (1 nM and 10 μM) reduces DNA fragmentation [21]. In this study, antioxidant pretreatment resulted in a reduction of DNA fragmentation by 2–5% in the presence of 0.6–2.5 μg/mL OTA. Schaaf and co-workers [29], in their studies on oxidative damage and free radical generation induced by OTA, observed that α-tocopherol at micromolar concentrations does not prevent the loss of cell viability in cultured renal cells. As described by Azzi [14], the effect of α-tocopherol depends on the oxidative environment in which it is active. This interaction allows α-tocopherol to act as a sensor, monitoring the cellular environment through concentration changes and transferring the information from the membrane to the nucleus. The effects of α- tocopherol on protein kinase C and the other molecular signaling pathways, as suggested by Fazzio *et al.* [30], could vary, considering the cell-specific pathways of cellular proliferation in which vitamin E can act.

## 3. Materials and Methods

### 3.1. Chemicals

Ochratoxin A, racemic α-tocopherol, penicillin, streptomycin and 3-(4,5-dimethylthiazol-2-yl)-2,5-diphenyltetrazoliumbromide (MTT) were purchased from Sigma-Aldrich (St. Louis MO, USA). Dulbecco’s Minimum Essential Medium (DMEM) and glutaMax were obtained from Gibco (Invitrogen); and FBS (South America) from Bio Whittaker. Ochratoxin A was dissolved in methanol to obtain a stock solution of 5000 μg/mL. According to the data obtained in preliminary studies [31,32], racemic α-tocopherol was dissolved in absolute ethanol to prepare a stock solution of 10 mM. These stock solutions were used for further dilutions in DMEM containing 0.6% FBS to obtain the final concentrations of each compound.

### 3.2. Cell culture

In this study, primary porcine fibroblasts isolated from embryo and from ear were used. Fibroblasts were grown in monolayers in 75-cm^2^ plastic culture flasks (Nunclon, Nunc Denmark) in Dulbecco’s Minimum Essential Medium (DMEM) supplemented with 1% glutaMax, 2500 I.U./mL penicillin, 2.5 mg/mL streptomycin and 10% FBS. Primary fibroblasts were cultivated in a humidified atmosphere with 5% CO_2_ at 37 °C and split 1:4 once a week. In all experiments in this study, we used embryonic porcine fibroblasts between passages 5 and 10 and ear between 5 and 8. All experiments were conducted in media containing 0.6% serum, the minimum useful concentration to maintain the selected *in vitro* models.

### 3.3. Determination of the half-lethal concentration and LDH release induced by ochratoxin A

Cells were seeded in 96-well culture plates (3000 cells/well, in 200 μL of complete medium) and were cultured for 24 h. A dose–response experiment was set up. Fibroblast cultures were exposed to increasing concentrations of OTA (0–10 μg/mL) for the following 24 or 48 h.

The effects of OTA treatments on fibroblast viability were determined using the MTT test. Cellular viability was determined using a colorimetric assay based on the production of the chromophore formazan from 3-(4,5-dimethylthiazol-2-yl)-2,5-diphenyltetrazoliumbromide (MTT). Formazan is produced in viable cells by the mitochondrial enzyme succinate dehydrogenase.

At the end of the incubation, the media were removed, the monolayers were washed with PBS twice, 150 μL MTT stock solution (5 mg/mL) in PBS was added to each well, and the plates were incubated for 3 h at 37 °C in a humidified chamber. To dissolve the formazan, 150 μL dimethyl sulfoxide was added to each well, after discharging the MTT solution. Absorbance at 540 nm was determined on a Biorad 680 microplate reader (Bio-rad, Veenendaal, The Netherlands).

Cells incubated with culture medium alone, representing 100% viability, were included as negative controls in all experiments.

The percentage cytotoxicity was calculated as follows:

Percentage cytotoxicity = (1 – mean optical density in presence of OTA/mean optical density of negative control) × 100. From these data the half-lethal concentration (LC_50_) of OTA for each primary cell culture was calculated.

Cell membrane damage induced by OTA was detected by LDH release using a CytoTox 96^®^ Non-Radioactive Cytotoxicity Assay (Promega) as instructed by the manufacturer.

Primary porcine fibroblasts were seeded in 96-well plates at the density as described above and cultured for 24 h. Afterward, the cells were exposed to OTA solutions (concentration range: 0–10 μg/mL) for 24 or 48 h. At the end of the incubation period the media were removed and cells washed with PBS twice.

LDH is a stable cytosolic enzyme released upon cell lysis. The amount of LDH was measured with an enzymatic assay using tetrazolium salts in conjunction with diaphorase. Briefly, after treatments supernatants were removed and centrifuged for 5 min at 1500 × g at 4 °C. 50 μL of each supernatant was transferred to a 96 well plate. Cells were lysed by adding 15 μL of 9% Triton X-100 solution in water per 100 μL of culture medium containing 0.6% of serum, followed by incubation for 1 h at 37 °C. Cells debris were removed by centrifugation for 5 min at 1500 × g at 4 °C and 50 μL of each sample was transferred to 96 well plate. Then, 50 μL of LDH substrate was added to the supernatants and cell lysates. After incubation for 30 min at room temperature in the dark, the enzymatic assay was stopped by adding 50 mL of 1 M acetic acid and the plate was read at 490 nm using a microplate reader.

The percentage of LDH release was calculated as the amount of LDH in the supernatant over total LDH from both supernatant and cell lysate.

### 3.4. Detection and quantification of DNA damage induced by ochratoxin A

DNA fragmentation was measured with the diphenylamine method as described by Sandau *et al.* [33]. Primary porcine fibroblasts were seeded in 75-cm^2^ flasks (density: 0.55 × 10^5^ cells/mL) and grown at 37 °C in 5% CO_2_ for 5 days. At sub-confluence, fibroblasts were incubated with OTA (0–10 μg/mL) for 24 h. After incubation, the media were removed and centrifuged at 1,800 × g for 20 min to collect the detached cells (fraction S). The fraction S was subsequently lysed in 5 mL of ice-cold lysis buffer [10 mM Tris, 1 mM EDTA (pH 8.0), 0.5% Triton X-100] for 30 min at 4 °C. The remaining adherent cells were scraped off the plastic and lysed in 5 mL of ice-cold lysis buffer as well. 

After cell lysis, the intact chromatin (fraction B) was separated from DNA fragments (fraction T) by centrifugation for 20 min at 13,000 × g. Samples were treated with one volume of 25% trichloroacetic acid (TCA), precipitated overnight at 4 °C, and recentrifuged 20 min a 13,000 × g at 4 °C, and then the supernatants were removed.

DNA was hydrolysed by adding one volume of 5% TCA to each pellet and heating 15 min at 90 °C in a heating block. DNA content was quantitated using the diphenylamine reagent. Afterward, 1600 μL of DPA solution (in 10 mL glacial acetic acid: 150 mg diphenylamine, 150 μL H_2_SO_4_ and 50 μL acetaldehyde 16 mg/mL solution) were added to each fraction and incubated for 4 h at 37 °C in the dark.

The OD_600_ of each fraction (S, B, and T) was determined. The percentage of DNA fragmented was calculated as the ratio of the DNA content in the supernatant (T) to that in the pellet (B), considering also the quantity released by cells undergoing apoptosis and lysis during the experiment.

Percentage of fragmented DNA = [(S + T)/(S + T + B)] × 100

### 3.5. Detection of DNA damage induced by ochratoxin A byTUNEL (TdT-mediated dUTP nick end labeling) assay

Primary porcine fibroblasts were seeded at a density of 0.4 ×10^5^ cells/mL in chamber slides, which had two chambers (Nunc Lab-Tek, Nunc, Denmark), which were previously coated with a thin layer of poly-L-lysine 0.01% (Sigma) to support cellular adhesion. Cells were cultured for 24 h in complete medium. Afterward, the media were removed and the fibroblast monolayers washed twice with PBS. Based on results obtained from previous assays, 0.6 μg/mL OTA and the appropriate LC_50_ (embryonic LC_50_: 5 μg/mL OTA; ear LC_50_: 1.2 μg/mL OTA) were added to each chamber slide for the following 24 h.

At the end of the incubation, the media were removed and the cells fixed with 4% paraformaldehyde at room temperature for 25 min. The TUNEL assay was performed using the DeadEnd Colorimetric Apoptosis Detection kit (Promega, Madison, WI, USA). The monolayers were washed twice with PBS and permeabilized by immersing the slides in 0.2% Triton X-100 solution in PBS for 5 min at room temperature. After washing with PBS, cells were incubated with biotinylated nucleotide mixture together with terminal deoxynucleotidyl transferase enzyme. Horseradish peroxidase–labeled streptavidin (streptavidin HRP) was then added to bind to these biotinylated nucleotides, which are detected using the peroxidase substrate hydrogen peroxide and the stable chromogen diaminobenzidine (DAB). Afterward, to visualize and estimate the apoptotic and normal cells, haematoxylin staining was performed. Images (20X and 40X) were captured under an Olympus BX51 microscope. For each experiment, ~500 cells were counted in randomly selected fields, and the percent of TUNEL-positive cells was calculated.

### 3.6. Determination of the effect of α-tocopherol against OTA-induced toxicity

To evaluate the most suitable concentrations of α-tocopherol for interaction experiments with OTA, a dose–response curve for this compound was established using serial concentrations from the nanomolar to micromolar range (data not shown). Cell culture setup and conditions were as detailed above. Primary porcine fibroblasts were cultured with LC_50_ doses of OTA in the presence or absence of α-tocopherol (1 nM or 1 μM). Cell viability and LDH release after antioxidant treatment were assessed as previously described.

To determine cell viability, primary fibroblasts were first pre-incubated for 3 h with α-tocopherol and then exposed to increasing concentrations of OTA for 24 or 48 h. Cells were also exposed to antioxidant alone or ethanol (the α-tocopherol solvent) alone to evaluate any non-specific effects.

Inhibition of cytotoxicity was determined by MTT, LDH release, DNA fragmentation and TUNEL assays and calculated as the percentage inhibition (percentage cytotoxicity OTA–percentage cytotoxicity of (OTA + antioxidant)).

### 3.7. Statistical analysis

The data are expressed as means ± standard errors (SE). At least three replicates at each incubation time were performed, and all the experiments were performed twice. Obtained data were analyzed by one-way ANOVA (General Linear Models Procedure) [34]; Duncan’s post-hoc multiple range test was used, with P ≤ 0.05 considered statistically significant.

## 4. Conclusions

Primary porcine fibroblast cultures offer new *in vitro* opportunities to study OTA cytotoxicity and the role of α-tocopherol in counteracting the several types of damage induced by this mycotoxin. OTA cytotoxicity developed through several mechanisms of action. The cellular inhibition associated with the LDH release and DNA fragmentation induced by OTA showed different sensitivities in the two fibroblast cultures. α-Tocopherol treatments could reduce the damage induced by OTA at different cellular levels. Our results point to the conclusion that the use of α-tocopherol offers new strategies to reduce OTA cytotoxicity, supporting its defensive role in the cell membrane and its multiple functions in cellular metabolism.
